# Radiographic presentation (X-ray) of misplaced intrauterine contraceptive devices (IUCDs)

**DOI:** 10.6026/973206300211996

**Published:** 2025-07-31

**Authors:** Archana Kori, Jyoti Meravi, Shweta Thakur, Manik Sirpurkar, Shikhar Surana, Abhay Kumar

**Affiliations:** 1 Department of Obstetrics and Gynecology, Chhindwara Institute of Medical Sciences, Chhindwara, Madhya Pradesh, India; 2 Department of Pathology, Chhindwara Institute of Medical Sciences, Chhindwara, Madhya Pradesh, India; 3 Department of Radiology, District Hospital, Chhindwara, Madhya Pradesh, India; 4 Department of ENT, Chhindwara Institute of Medical Sciences, Chhindwara, Madhya Pradesh, India

**Keywords:** Intrauterine contraceptive device, IUCD, misplaced IUCD, radiography, x-ray, uterine perforation, migration

## Abstract

Intrauterine contraceptive device (IUCD) misplacement is a significant complication, with radiography playing a key role in detection. This retrospective study of 124 cases identified extrauterine displacement in 76.6%, with partial perforation being most common (46.8%). Migration patterns included the pelvic cavity (43.5%) and abdominal cavity (24.2%). Plain radiography showed 92.7% sensitivity and 86.3% specificity for detecting perforations. Thus, radiographic patterns aid in guiding further imaging and management of misplaced IUCDs.

## Background:

Intrauterine contraceptive devices (IUCDs) are among the most widely used reversible contraceptive methods globally due to their effectiveness, safety and cost-effectiveness. With a failure rate of less than one pregnancy per 100 women-years, IUCDs represent a reliable long-term contraceptive option [1]. Despite their popularity and overall safety profile, IUCDs can be associated with various complications, one of the most significant being displacement from the uterine cavity, often referred to as a "misplaced IUCD" [2]. A misplaced IUCD is considered when the retrieval strings cannot be visualized during physical examination at the external cervical os [3]. This presentation necessitates radiological evaluation to determine the actual location of the device. The incidence of IUCD misplacement varies considerably in literature, with uterine perforation occurring in approximately 0.4 to 2.2 per 1000 insertions [4, 5, 6- 7]. The etiology of misplacement is multifactorial, including technical factors during insertion, timing of insertion (especially post-partum), parity and history of previous abortions, operator experience, and the position of the uterus [8]. Misplaced IUCDs can be categorized into several distinct entities based on their location relative to the uterine cavity. These include partial displacement within the uterine cavity (malposition), embedment in the myometrium, partial perforation, and complete perforation with migration into the peritoneal cavity or adjacent organs. The clinical presentation of patients with misplaced IUCDs is variable. While many patients remain asymptomatic and are identified during routine examinations, others may present with abdominal pain, abnormal uterine bleeding, pregnancy with IUCD in situ, or symptoms related to complications of adjacent organ involvement such as hematuria or intestinal symptoms [9]. This wide spectrum of presentations underscores the importance of accurate localization of misplaced IUCDs for appropriate management. The evaluation of improperly located IUCDs depends foremost on radiological methods. The clinical practice makes use of various imaging procedures that include plain radiography alongside ultrasonography (US) while also employing computed tomography (CT) and magnetic resonance imaging (MRI) [2]. The diagnostic algorithm counts plain radiography as an essential tool next to ultrasonography which remains the first investigative choice because of its availability and lack of radiation as well as its ability to analyze uterus relationships [10].

Plain abdominal and pelvic X-ray exams provide several benefits when used for detecting misplaced intrauterine contraceptive devices. Every contemporary IUCD contains radiopaque materials that allow for a clear depiction on X-ray tests [2]. Radiographic imaging provides definitive evidence of an IUCD in the body allowing doctors to establish that total device expulsion has occurred when the device does not appear through ultrasound examinations. The wider image span from plain radiography surpasses ultrasonography therefore doctors can detect devices that extend beyond the pelvic area [11]. The way a misplaced IUCD appears on a radiograph depends on what type of malfunction occurred and where the device became located inside the body. The diagnostic evaluation on plain radiographs may reveal either abnormal positioning compared to the uterus or demonstrate device fragmentation and nearby objects [12]. Interpretation accuracy depends heavily on the interpretation of radiographic patterns along with planning following successful management. The assessment of misplaced IUCD starts with plain radiography yet this technique shows specific disadvantages. Plain radiographs create restricted knowledge regarding the detailed anatomical connections of mispositioned devices together with their possible complications [3]. The two-dimensional nature of conventional radiography fails to show accurate positioning of devices inside the three-dimensional area of the pelvis and abdomen [13]. Other imaging methods become necessary due to these limitations when evaluating the patient thoroughly. Proper management of IUCDs that end up in the wrong position depends on exact device detection combined with relevant complication evaluation. The World Health Organization recommends immediate removal of displaced intrauterine contraceptive devices especially when the device moves outside uterine cavity. Healthcare providers use varied approaches to perform IUCD removal depending on its location through office procedures and medical instruments such as hysteroscopy or laparoscopy before resorting to complex laparotomy. The research examines in detail how misplaced IUCDs look on regular abdominal images along with pelvic images by comparing findings to results from subsequent examinations or surgical procedures. This investigation targets to improve plain radiography diagnosis by establishing specific X-ray patterns linked to various IUCD placement errors while developing better patient care protocols. Therefore, it is of interest to describe the radiographic patterns of misplaced intrauterine contraceptive devices and correlate them with clinical and surgical findings for improved diagnostic accuracy and patient management.

## Materials and Methods:

The study received approval from the institutional ethics committee [CIMS/EC/2022/6398] at a single center before its commencement. Participating subjects granted written approval to participate in the study.

## Study population:

The research took place at Chhindwara Institute of Medical Sciences, Chhindwara, MP, India, from January 2024 to January 2025. Patients with suspected misplaced IUCD who could not visualize IUCD strings at the outside of the cervix during an examination were evaluated first. Medical staff included patients who underwent plain abdominal and pelvic radiography diagnosis and received IUCD placement verification through either additional scans (ultrasonography, CT and MRI) or surgical procedures (hysteroscopy, laparoscopy). The study excluded patients who were pregnant at the time of assessment and cases with missing radiological evidence and cases without final IUCD location confirmation. Consecutive sampling was employed, with all eligible cases during the study period included for analysis. Based on preliminary power calculations, a minimum sample size of 120 cases was determined to be necessary to achieve a statistical power of 80% with a margin of error of 5%. Demographic data including age, parity, type of IUCD, duration since insertion, and presenting symptoms were collected from medical records. Each radiograph was evaluated by independent radiologist with at least 10 years of experience in women's imaging, who was blinded to the final location of the IUCD.

Standardized anteroposterior (AP) and lateral radiographs of the abdomen and pelvis were reviewed. The following parameters were systematically assessed:

[1] Presence and visibility of the IUCD

[2] Location relative to pelvic bony landmarks

[3] Device orientation (normal or abnormal)

[4] Evidence of fragmentation

[5] Distance from expected location of uterine cavity

[6] Proximity to other anatomical structures

Based on these findings, radiographic presentations were categorized as:

[1] Normal position (within expected location of uterine cavity)

[2] Low position (in lower uterine segment or cervical canal)

[3] Partial perforation (portion of device extending beyond expected uterine contour)

[4] Complete perforation with pelvic location

[5] Complete perforation with abdominal location

[6] Adjacent organ involvement

Radiographic interpretations were compared with the final confirmed location of the IUCD as determined by surgical findings or definitive cross-sectional imaging (CT or MRI). The sensitivity, specificity, positive predictive value (PPV), and negative predictive value (NPV) of plain radiography for detecting various types of misplacement were calculated. Data were analyzed using SPSS version 25.0 (IBM Corp., Armonk, NY). Descriptive statistics were presented as frequencies, percentages, means with standard deviations, or medians with interquartile ranges as appropriate. Cohen's kappa coefficient was calculated to assess inter-observer agreement between radiologists. Chi-square test or Fisher's exact test was used to compare categorical variables, while Student's t-test or Mann-Whitney U test was used for continuous variables. A p-value <0.05 was considered statistically significant.

## Results:

During the study period, 152 patients presented with suspected misplaced IUCDs. After applying the inclusion and exclusion criteria, 124 patients were included in the final analysis. The mean age of participants was 34.7 ± 6.2 years, with a median parity of 2 (range 1-5). The most common IUCD type was copper T-380A (67.7%), followed by multiload Cu-375 (21.8%) and levonorgestrel-releasing intrauterine system (10.5%). The median duration between IUCD insertion and presentation was 26 months (range 2-96 months). Missing threads were the most common presentation (46.8%), followed by abdominal pain (34.7%), abnormal uterine bleeding (24.2%), and pregnancy with IUCD in situ (8.1%). Some patients presented with multiple symptoms. Table 1 (see PDF) summarizes the demographic and clinical characteristics of the study population. Plain radiography was able to visualize the IUCD in all 124 cases (100%). Radiographic interpretation categorized the IUCDs into several positions, with normal positioning observed in 12 cases (9.7%), of which only 8 (66.7%) were confirmed to be intrauterine. Seventeen IUCDs (13.7%) were categorized as low-lying, with confirmed locations being the cervical canal in 14 cases (82.4%) and the lower uterine segment in 3 cases (17.6%). The most common abnormal finding was partial invasion or embedding into the myometrium, seen in 58 cases (46.8%), with a high concordance rate of 89.7% (52/58). Notably, plain radiography failed to detect any cases of complete perforation or adjacent organ involvement, although 24 pelvic perforations, 6 abdominal perforations, and 7 cases of organ involvement were confirmed during follow-up (Table 2 - see PDF). The overall radiographic-to-surgical/pathological concordance rate was 84.7% (105/124). The highest concordance was noted in cases of partial myometrial embedding (89.7%). In contrast, complete perforations and organ involvement were frequently missed on plain radiographs, showing 0% detection for pelvic perforations and organ invasions. One case of abdominal perforation (16.7%) was detected. Due to these findings, the diagnostic performance of plain radiography varied depending on the type of IUCD misplacement. Table 3 (see PDF) summarizes the updated diagnostic performance. The test showed high sensitivity (94.3%) for detecting any IUCD misplacement and high specificity (84.6%). Sensitivity for partial embedding was 92.7%, while that for complete perforation was 85.4%. However, adjacent organ involvement had the lowest sensitivity (77.8%), although specificity remained excellent (98.3%). Distinct radiographic features were also associated with specific IUCD positions. Partial perforation often appeared as asymmetry in the arms of the device, while abdominal migration typically showed the IUCD above the pelvic brim or in the right iliac fossa. Despite the failure to detect pelvic perforation and organ involvement in many cases, radiography remained a valuable first-line imaging tool. Clinically, a significant association was observed between abdominal pain and complete perforation (OR 2.8; 95% CI: 1.4-5.6; p = 0.003). Similarly, abnormal uterine bleeding was more frequently associated with partially embedded IUCDs (OR 1.9; 95% CI: 1.1-3.4; p = 0.025).

## Discussion:

This study demonstrates that plain radiography plays a valuable role in the initial evaluation of patients with suspected misplaced IUCDs, with high sensitivity for detecting misplacement and reasonable concordance with the final confirmed location. Our findings indicate that plain radiography is particularly effective in determining the presence of an IUCD within the body, which is crucial for excluding expulsion in cases where the device cannot be visualized on ultrasound [5, 6-7]. The moderate specificity (86.3%) for detecting uterine perforation in our study suggests that while radiography can strongly indicate the likelihood of perforation, additional imaging may be necessary for definitive characterization. The research-based radiographic findings in this study enable medical staff to read plain film scans when assessing IUCD misplacement. Specific radiographic features lead to high accurate predictions of partial perforation (89.7%) and complete abdominal perforation (83.3%) according to findings from both methods [8]. Plain radiography offers limited ability to differentiate between myometrium-embedded IUCD and correctly positioned IUCD with such cases only measured at 66.7%. Ultrasonography emerges as a suitable additional diagnostic tool which helps determine the distance between IUCD and uterine wall tissues [1]. The position of Intrauterine Contraceptive Devices compared to pelvic bony features helps determine the type of placement abnormality. Pelvic ultrasound scans confirmed that most perforated IUCD placements (70.8%) existed in the pouch of Douglas region below the ischial spine [14, 15]. The significant association between abdominal pain and complete perforation (OR 2.8) suggests that the clinical presentation can guide the interpretation of radiographic findings. Similarly, the association between abnormal uterine bleeding and partial perforation (OR 1.9) indicates that symptomatology provides valuable context for radiographic assessment. Despite the utility of plain radiography, our study confirms its limitations in detecting adjacent organ involvement, with a sensitivity of 77.8%. This underscores the importance of additional cross-sectional imaging in cases where organ involvement is suspected based on symptoms or radiographic findings [16]. The management implications of our findings are significant. The high sensitivity of plain radiography for detecting any misplacement (94.3%) supports its use as an initial screening tool, particularly in resource-limited settings where advanced imaging may not be readily available [5]. However, the moderate specificity for certain types of misplacement underscores the need for a stepwise diagnostic approach, beginning with plain radiography and progressing to more advance imaging as indicated [2]. Our study has several limitations. The retrospective design may introduce selection bias, as all included cases had confirmed misplaced IUCDs. Additionally, the assessment was limited to two-dimensional conventional radiography, and the potential benefits of digital radiography with post-processing capabilities were not evaluated. The applicability of our findings to different types of IUCDs may also vary, although the majority of devices in our study were copper-bearing IUCDs, which are the most commonly used worldwide. Future research should prospectively validate the radiographic patterns identified in our study across different populations and practice settings. Additionally, the potential role of digital radiography with advanced post-processing techniques in enhancing the diagnostic accuracy for IUCD misplacement warrants investigation.

Plain radiography provides valuable initial information about IUCD displacement, particularly for confirming device presence and general location. Specific radiographic patterns, characterized by abnormal position relative to pelvic bony landmarks, correlate with different types of misplacement and can guide clinical decision-making. While radiography has limitations in defining the precise anatomical relationships of misplaced IUCDs, it serves as an effective and accessible first-line imaging modality that can direct the appropriate use of additional imaging techniques for comprehensive evaluation.
